# T-Patterns Revisited: Mining for Temporal Patterns in Sensor Data

**DOI:** 10.3390/s100807496

**Published:** 2010-08-10

**Authors:** Albert Ali Salah, Eric Pauwels, Romain Tavenard, Theo Gevers

**Affiliations:** 1 Informatics Institute, University of Amsterdam, Science Park 107, 1098 XG, Amsterdam, The Netherlands; 2 CWI, Science Park 123, 1098 XG, Amsterdam, The Netherlands; E-Mail: eric.pauwels@cwi.nl; 3 University of Rennes 1 - IRISA, 35042 Rennes Cedex, France; E-Mail: romain.tavenard@irisa.fr

**Keywords:** sensor networks, temporal pattern extraction, T-patterns, Lempel-Ziv, Gaussian mixture model, MERL motion data

## Abstract

The trend to use large amounts of simple sensors as opposed to a few complex sensors to monitor places and systems creates a need for temporal pattern mining algorithms to work on such data. The methods that try to discover re-usable and interpretable patterns in temporal event data have several shortcomings. We contrast several recent approaches to the problem, and extend the T-Pattern algorithm, which was previously applied for detection of sequential patterns in behavioural sciences. The temporal complexity of the T-pattern approach is prohibitive in the scenarios we consider. We remedy this with a statistical model to obtain a fast and robust algorithm to find patterns in temporal data. We test our algorithm on a recent database collected with passive infrared sensors with millions of events.

## 1. Introduction

Endowing environments with a capability to respond intelligently to different situations depends on observing the activity in the environment and deriving patterns of behaviour. For instance in ubiquitous environments, a wealth of sensor data is produced by observing the behaviours and interactions of humans. Mining the data for temporal patterns aims to discover associations and structure, either in an offline manner to pave the way for new designs and applications, or in an online manner to ensure adaptation of the environment to the users.

Two things make this task especially challenging. First of all, in a real environment, action patterns that are composed of separate events are interleaved, either by the presence of multiple factors that act on the environment (such as multiple users triggering different sensors), or by the presence of single actors performing multiple actions at the same time. Thus, taking an event window to predict the next event in the system will simply not work. Secondly, these patterns exist in different time intervals, and the time difference between related events of a single action can have a large variation. Consequently, detecting associations with these patterns becomes a very challenging task, and most traditional pattern analysis methods are not directly applicable.

In this paper, we review approaches to the problem of detecting temporal patterns and extend the T-Pattern algorithm [1,2], which was successfully applied for detection of temporal patterns in behavioural sciences. The original T-pattern algorithm has quadratic time complexity in the number of sensors, as well as in the number of discrete time steps considered for pattern search (*i.e.*, event horizon). We show how this complexity can be reduced, and propose a modified algorithm that is quadratic only in the number of sensors. We also discuss the application of Bonferroni correction to reduce spurious patterns. Our extensions allow the application of the modified algorithm to broader settings. We test our approach on simulated and real datasets.

This paper is structured as follows: In Section 2, a detailed survey of the relevant literature is presented. The T-pattern method and our proposed modifications to it are presented in Section 3 and Section 4, respectively. In Section 5, we test our methodology on a simulated database and on the recently collected Mitsubishi Electric Research Laboratories (MERL) motion detection database [3]. We conclude in Section 6.

## 2. Description of the Problem and Related Work

The application of sequential pattern recognition in sensor networks includes long-term environmental monitoring [4], alarm event detection and propagation [5,6], localization and tracking of objects [7–9], recognition of human behaviour and interactions [10–16], and intelligent resource management [17] among others. The temporal data are in the form of a sequence of events, derived from a number of sensors that may have different modalities. While some papers make the simplifying assumption that a single cause is behind all events (e.g., in the home of an elderly person [15]), the real challenge of the problem is the existence of multiple causes, triggering unrelated events one after the other. In this paper we will describe an approach to deal with multiple causes.

In sensor-based applications it is possible to view the physical causes of measured sensor events as hypotheses, for which a belief can be expressed via Bayesian techniques [18]. Simple smoothing methods like Kalman filters are usually inadequate for most real applications, since there are usually multiple hypotheses to be taken into consideration and linearity assumptions are overly simplistic. In [18] particle filters were proposed to represent multiple probabilistic hypotheses about the factors simultaneously, in a location estimation application.

There are several algorithms that can potentially discover patterns of event sequences. An early approach was proposed by [19], which seeks to find sequence generating rules that will produce a set of plausible events after each event. While it is possible to treat the sequence of events as a string of symbols and look for patterns, it makes more sense to consider the temporal dimension of these events, where the time lag between the events plays an important role. Some algorithms indeed take this information into account. For instance in recurrent neural networks, the temporal dimension is modelled with the help of context units [20]. However, recurrent neural networks and related approaches cannot deal with overlapping patterns, they quickly become cumbersome for larger input intervals, and they require lots of training samples.

Markov models have been recently employed to tackle simplified versions of this problem, where there are no action overlaps, and events are generated as one long sequence [21]. These models have three main disadvantages for the problem at hand. First and foremost, the first order Markovian assumption may not hold, as action patterns are construed as sequences of events, and the complete sequence is relevant for the prediction of the next event. Secondly, the estimation algorithms assume that the topology of the HMM-structure is known, which is not the case. Finally, they cannot predict patterns that have long event intervals. Other spatio-temporal learning paradigms that rely on state transitions include dynamic time warping [22], and finite state machines [23].

The problem of detecting interesting sequences of events in temporal data has been explored in the data mining literature [24–30], but these methods are rarely applied to real-time analysis of sensor data. In [27] the WINEPI algorithm is proposed, where a fixed-length temporal window is used to filter out cohesive episodes, followed by a threshold-based selection. The length of the temporal window is chosen by the user, and controls the proximity of events that will be associated with each other in a given episode. A shortcoming of the approach is that shorter event sequences will be more favoured, which can be alleviated by increasing the window length proportionally to the episode length [31] or by introducing other measures of interestingness that will select longer episodes over short ones [32]. In a series of studies closely related to ours in terms of interval usage, temporal pattern mining was applied to discover sequential patterns in object tracking sensor networks [8,9]. A multi-level hierarchical structure was adapted to facilitate access to the stored pattern database, and thus allow real-time tracking of objects. The idea of modeling event timings with Gaussian mixtures was used in Micheloni *et al.* for a surveillance application, but in their work the inter-event relations are hand-crafted through a finite state machine representing the events, as the focus is in detecting anomalous events [14].

A recent approach involves PCA-based methods to uncover daily human behaviour routines [33]. The data for each subject are stored in an activity matrix, whose most prominent eigenvectors (dubbed *eigenbehaviors*) are then interpreted. One obvious drawback with this method is that it requires a fixed sized activity vector. Additionally, there is no hierarchical decomposition of activities.

Finding a *dictionary* of patterns is possible with compression-based algorithms that treat events as “words” in a stream, and seek the patterns that lead to the best compression of the stream. These methods use the Lempel-Ziv compression algorithm, which is known to achieve Markov entropic compression, or a variant of it (e.g., Lempel-Ziv-Welch and Active Lempel-Ziv algorithms) [34].

The basic Lempel-Ziv algorithm (LZ78) uses an automatically updated dictionary to extract recurring “words” (patterns) in a string. It constructs a symbol tree, where the paths from root to leaves constitute the words in the dictionary. The Lempel-Ziv-Welch (LZW) variant starts off with a pre-defined basic dictionary (in the case of sensor networks these are single sensor events) to avoid ill-detected patterns at the beginning of the stream and to introduce some continuity. The Active LeZi (ALZ) uses a sliding window of length *l* (length of the longest phrase in LZ table) on the stream to extract all possible sequences of size *l*.

LZW and Active LeZi both aim at adding continuity to LZ pattern extraction, yet they still have linear complexity, which is a beneficial feature for a real-time event detection system. On the other hand, none of the compression based methods take into account the temporal structure of the patterns, as the time delays are not modelled, and subsequently overlapping events may escape detection. For a dense, low-cost sensor network without the identification of event source, this is a major drawback [2]. This is the main reason why we turn our attention to *T-patterns* as discussed in the next section.

## 3. T-patterns

Most of the temporal pattern detection methods mentioned in the related work section cast the problem into a simpler representation by retaining only the order of events, and look for repeated patterns. In neural network, HMM, and compression based approaches, the emphasis is on predicting the next event, which is not a suitable perspective for an environment where multiple people trigger sensors, and the sensor patterns that follow a logical order (*i.e.*, triggered by one person during an activity) may be interleaved. Furthermore, the actions that cause sensor events can have different typical interval lengths, which makes representations with fixed temporal extent unwieldy.

In the *T-pattern* approach, as introduced and explored by Magnusson, symbolic time series are investigated, where each symbol represents the onset of a particular event or activity, with the principal goal of elucidating possible relationships between pairs of symbols and then building trees of temporal dependencies in a hierarchical fashion [1]. A thorough search is conducted on the training sequence for symbols of an ever-growing dictionary. As the algorithm proceeds, pairs of strongly correlated events are joined into new events, and the search is resumed with the expanded dictionary.

To recast this problem in a mathematical framework, we first introduce some notation. We denote by *A* = (*A*_1_, *A*_2_,…, *A_n_*,…) the (ordered) sequence of times at which an A-event occurs. For the inter-event time-intervals we use the notation *T_A_*; more precisely: *T_A_*(*n*) = *A_n_* − *A*_*n*−1_. Similar notation is used for B-events. Since we need to find out whether A-events tend to induce B-events, we refer to the combination of an A-event and the *first* subsequent B-event as an AB-event. The time-interval separating these two events is denoted by *T_AB_*. More formally:

$$T_{\mathrm{AB}}(k)=B_{k*}-A_{k}\;\;\text{where}\;\;k*=\text{arg}\;\text{min}\{j|B_{j}>A_{k}\}$$

Finally, we will denote by *T̃_B_* the time-interval between two successive B-events between which at least one A-event occurred. This definition means that the set of *T̃_B_*’s constitutes a subset of the *T_B_*-times, with a bias towards longer *T_B_*-values as short B-intervals are less likely to contain an A-event.

Magnusson introduced the notion of a *critical interval* (CI): [*d*_1_, *d*_2_] is considered to be a CI for the pair of symbols (events) (*A*, *B*) if the occurrence of *A* at time *t* entails that *B* is more likely to occur in the time interval [*t* + *d*_1_, *t* + *d*_2_] than in a random interval of the same size. He then suggests to use the standard *p*-value to gauge how exceptional the observed frequency of the combination under scrutiny is. More precisely, suppose the total data stream has length *T* with *N_A_* and *N_B_* occurrences of A and B, respectively. We assume (following Magnusson [1]) as null-hypothesis that A and B are independent Poisson processes with intensity (*i.e.*, the average number of events per unit time interval) λ*_A_* = *N_A_*/*T*, and λ*_B_* = *N_B_*/*T*, respectively. Now, given a CI [*d*_1_, *d*_2_] we find all the times *t_i_* at which an A-event occurs and then cumulatively collect the B-events that occur in the intervals [*t_i_* + *d*_1_, *t_i_* + *d*_2_], thus arriving at a number *N_AB_*. Notice that under the null-hypothesis, the expected number of B-events in a time interval of length *d* equals *μ_B_* = λ*_B_d*. In particular, the probability of not observing a B-event in this CI is therefore equal to *π*_0_ = *e*^−*μ*_*B*_^= *e*^−λ_*B*_*d*^. The above-mentioned *p*-value is then computed as the probability of observing at least *N_AB_* B-events in the CI, if we assume that A and B are independent. Hence,

$$\begin{array}{lll} p & = & P(N_{\mathrm{AB}}\;\text{B-events or more }|\text{A, B are independent)}\\ & = & 1-P(\text{strictly less than }N_{\mathrm{AB}}\;\text{B-events}|\text{ A, B are independent)}\\ & = & 1-\sum_{k=0}^{N_{\mathrm{AB}}-1}P(\text{exactly }k\;\text{B-events}|\text{A, B are independent})\\ & = & 1-\sum_{k=0}^{N_{\mathrm{AB}}-1}\left(\begin{array}{c} N_{A}\\ k \end{array}\right){\left(1-\pi_{0}\right)}^{k}\pi_{0}^{\left(N_{A}-k\right)}. \end{array}$$

Magnusson suggests, as a T-pattern detection scheme, to test, for every possible pair of symbols of the form (*A*, *B*), every possible CI, from the largest to the smallest one, until the *p*-value is sufficiently small indicating significance (0.005 is a typical upper bound). Note that *p* will be high for high values of *d*, which means that short intervals will be favored.

T-patterns were previously used in modeling complex interactions in behavioural studies and sports events [35], and the core algorithm is commercialized. A related software package, called *C-quence*, is developed by Duncan and Collier [36]. This rule-based algorithm allows searching for user-defined event sequences with varying inter-event timings.

## 4. The Modified T-Pattern Algorithm

We propose two modifications to the T-pattern algorithm to make it more resilient to spurious patterns, and to make the search for patterns more robust.

### 4.1. Testing Independence between Two Temporal Point Processes

The repeated significance expounded in the preceding section substantially increases the risk of false positives (suggesting spurious dependencies), since it increases the chances of finding random correlations between sensors. Applying a Bonferroni correction would be one way to mitigate this adverse effect. This can be done by replacing the *p*-value of significance testing by *p/α*, where *α* is the expected number of significance tests. However, this parameter cannot be known beforehand, as the pattern dictionary emerges incrementally. We will later discuss a way of estimating *α* that can be used in practice and report results with it. We now put forward a more efficient way of testing this independence between A and B, which is based on the following proposition.

**Proposition 1** *If A and B are independent temporal point processes, then*

$$T_{\mathrm{AB}}∼U(0,{\tilde{T}}_{B}).$$

This proposition asserts that if the A and B processes are independent, then whenever an A-event occurs between two successive B-events, it will be uniformly distributed in that interval. It is intuitively clear that non-uniformity of A within the B-interval would allow a keen observer to improve his or her prediction of the next B-event, thus contradicting independence. More formally, it is well-known that for a *single* Poisson process (e.g., the A-process) occurrences within a given time-interval are uniformly distributed (*i.e.*, if we know that a given interval harbours exactly *n* events, then these events will be uniformly distributed across that interval). If we accept that the B-process is independent of A, then it is legitimate to define the said interval as the time between two successive B-events. It therefore follows that the proposition holds for two independent Poisson processes (with constant intensity).

More complicated processes can be modelled by allowing the intensity to vary in time (*i.e.*, λ = λ(*t*)). One way of implementing a time-varying intensity is to start from a constant intensity process and locally squeeze or stretch the time axis (as dictated by the local value for λ(*t*)). If the inter-event times are small compared to the time scale at which λ(*t*) fluctuates, these local temporal deformations are well approximated by (locally) linear transformation and it is well-known that linear transformations preserve uniformity.

**Figure 1.**
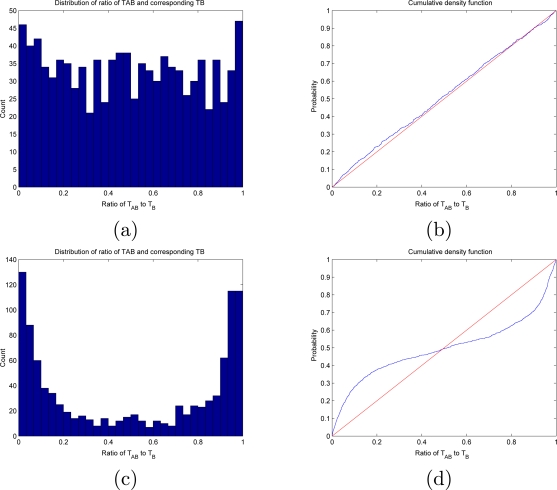
**(a)** Histogram of ratio *T_AB_*/*T̃_B_*, for independent processes A and B (sensor firings at physically distant locations, from the MERL dataset, see Section 5). **(b)** According to Prop.1, this ratio should be uniformly distributed between 0 and 1, a fact which is even more clearly borne out by plotting its cumulative density function against the theoretically predicted one. (The *p*-value in this case was 0.61, which means that the null hypothesis of independence is accepted.) **(c)** A similar histogram for two strongly correlated sensors (in physical proximity) and **(d)** the cumulative distribution function.

Proposition 1 therefore allows us to formulate a statistical procedure to test whether A and B are dependent: using the notation established above we compare for each event *A_k_* the time till the next B-event to the current B-interval length:

$$U(k)=\frac{T_{\mathrm{AB}}(k)}{{\tilde{T}}_{B}(k)}=\frac{B_{k*}-A_{k}}{B_{k*}-B_{k*-1}}$$

which, under the assumption of independence, should be uniformly distributed between 0 and 1: *U* ∼ *U* (0,1) (See Figure 1). This can be easily checked by any number of standard statistical tests (e.g., Kolmogorov-Smirnov). If the null hypothesis (independence) is rejected, then it makes sense to start looking for inter-event time intervals (*i.e.*, CI’s). This is taken up in the next section.

### 4.2. Modelling Inter-Event Times

The CI detection scheme as proposed in [1] exhaustively tests all possible intervals in search of possibly significant *p*-values. As pointed out earlier, this approach therefore risks to return lots of spurious results, leading to fallacious associations. Furthermore, the number of CI searches for a single pass of the T-pattern algorithm is *O*(*n*^2^*h*^2^), where *n* is the number of event types (or sensors) and *h* is the event horizon. Consequently, it is desirable to shorten this search if possible. We consider here two straightforward schemes that will reduce this time complexity:

**Shrinking interval T-Patterns (SITPat):** Suppose we are searching for a CI between events A and B, and the event horizon is given as [1, *h*]. The number of possible CIs is $\frac{h(h+1)}{2}$, which is why the original T-pattern scheme is quadratic in *h*. Now consider the following greedy approach. We start with the largest interval, [1, *h*], and shrink it from left and right, as long as the *p*-value decreases. The remaining interval [*i*, *j*] is once more shrunk from left and right, each time until a unit interval remains, to handle the case of two patterns that connect A and B. If we assume that the expected length of CI is half of the event horizon, the number of tests for a single pair of events is $\frac{3}{2}h$, thus linear in *h*.

**Tree-search T-Patterns (TTPat):** We first test the largest interval, [1, *h*], then proceed by splitting it into two and testing the left and right intervals. We select the branch with smallest *p*-value, and continue. This approach will require 2 log_2_(*h*) tests for a single pair.

**Figure 2.**
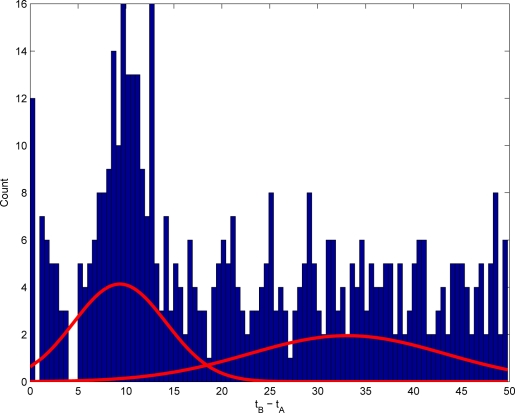
The distribution of the first B event after any A event. The mean and the standard deviation of the sharp Gaussian gives the critical interval for the A-B event.

Our proposed scheme (GMMTPat) has a complexity independent of *h*; it considers a single test per event pair at most. In this scheme, if the above-discussed uniformity test has rejected independence, then we look for the characteristic period by modelling the conditional probability *P*(*B* at *t* + Δ*t* | *A* at *t*) using Gaussian Mixture Models (GMM). More precisely, all the A-events are aligned at time zero, whereupon the first B-events are plotted. If an A-event tends to induce a B-event after a delay of *t* time-units, this will show up in this plot as a significant peak. All the non-related B-events will contribute to a very diffuse background. For that reason, we model the B-events as a 2-component GMM. One sharp and localized peak sits on top of the critical interval, while all the other B-events give rise to a flat and broad second component. The standard variation of the sharp peak immediately suggest a value for the width of the CI. Figure 2 illustrates this point.

**Figure 3.**
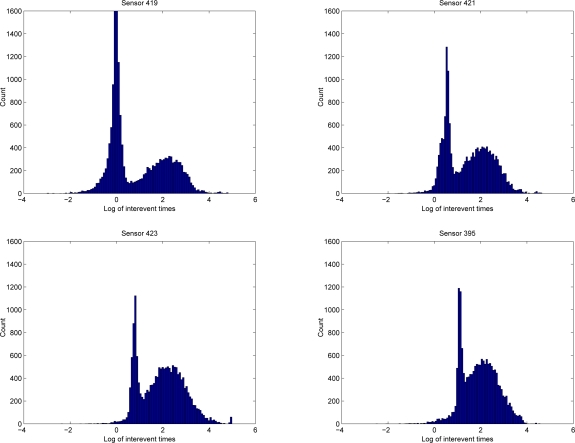
*Left to right, top to bottom:* Histogram of the logarithm of inter-event times (relative to firings of sensor 418) for four sensors along a MERL corridor (See Section 5). The sharp peak caused by people walking down the corridor and setting off the sensors in succession, is clearly seen as a gradual shift.

The peaks are even more pronounced if we plot the inter-event times against a logarithmic scale. We illustrate this on the MERL data, described in Section 5 Figure 3 shows such plots for time-intervals between the firing of sensor 418 and a subsequent firing of four other sensors along the same corridor for the MERL dataset. It transpires that there is a clear peak in the activity of sensor 419 after 10^0^ = 1 seconds, and this peak gradually shifts to a value of approximately 10^1^ = 10 seconds for sensor 395 which is several meters down the corridor.

## 5. Experiments

### 5.1. An Experimental Testbed

In order to compare the modified T-pattern approach with the original T-pattern scheme and compression based approaches, we have created a simple and realistic experimental setup by simulating a small number of interruption sensors in an office environment. A pre-defined event dictionary serves as a catalogue of prominent behaviours, where each behaviour takes the form of a number of sensor activations (events) separated by pre-defined time intervals. The events each correspond to some repeated activity, for instance going to the coffee machine, or to the photocopier. Depending on the layout and the working habits of the actors in the environment, there will be some consistent patterns, which our algorithm seeks to find. We create dummy office layouts with different sensor placements, and simulate activities of one or more users in them. Layout 1 is a rectangular office corridor block, with one door in the middle of each floor segment and sensors on the left and right hand side of three of these doors. Layout 2 consists of one entrance door connected to three corridors, and sensors placed along the corridors. Since the behaviour habits used in the simulation are known to us, we have the ground truth for the generated patterns. The existence of multiple users means that different sensors may be simultaneously activated, breaking the chain of causality (*i.e.*, prediction of the next event becomes very difficult).

These user-provided interval lengths are used in conjunction with an assumption of Gaussian noise between each triggered event. One or two users are simulated in the environment, where each user selects a behaviour from the dictionary, and executes it with a probability *e*. As the number of users is increased, the generated sequence of patterns gets more difficult to disentangle.

We have tested two event dictionaries with six sensors, and generated training and test sequences by simulating one or two persons. We have investigated to what degree we could use the patterns discovered in the training phase as predictors for events in the second stream. The prediction is made for each discrete time slot, which is more granular than just predicting the next event. We have contrasted compression based methods, T-patterns, and our modified T-pattern approach. As the first symbol emitted by each new pattern is random and therefore completely unpredictable, and as individual patterns are short, the prediction rate will have an inherent upperbound.

We have associated probabilistic confidence values with each prediction. The compression-based approaches look at their prediction tree, and compute a posterior probability for each possible event. These probabilities are normalized to sum up to unity, and they indicate a confidence in the prediction; if the context is weak, the posteriors of several events will be close to each other, whereas a strong prediction is evident in a strong posterior. By setting a threshold of confidence (*i.e.*, the minimum value the highest posterior should take for a prediction claim), we will reduce the number of overall predictions, but increase the overall accuracy. We note here that the compression methods have no tunable parameters, and repeat that the sliding window size in ALZ is automatically set to the length of the longest phrase in LZ table. The size of the dictionary grows sublinearly with the size of the input for each compression method.

For the T-patterns and the GMM T-patterns, the critical intervals are taken into account. Ordinarily, the stored patterns are useful for predicting the occurrence of multiple events in overlapping time intervals in near future. This result is more informative than the compression-based algorithm predictions. However, to make their comparison possible, we supply the algorithm with the time that an event occurs, and require the prediction of the event type. For this purpose, all detected T-patterns in the pattern dictionary are used to create their critical intervals based on a fixed history, and these are checked for inclusion of the event time. For each applicable pattern, a uniform distribution within the critical interval is assumed, and the probabilities of different patterns are combined.

We summarize the experimental results in Table 1. It was obtained by computing for each experiment the correct prediction rate for a confidence level of 20%. The significance of the proposed improvements is obvious. Table 1 shows that for a given layout and a single user in the environment, compression based methods have good prediction accuracy (within the limits imposed by layout and behaviour dictionary). For Layout 2, ALZ achieves 66.4 per cent prediction, where the perfect predictor would achieve 70 per cent. This shows that the parameters of the compression methods are correctly set. In fact, these methods have few tunable parameters, and they are robust in face of these. What is important here, is that the compression methods deteriorate rapidly as soon as multiple users are introduced into the environment, as it is evident from the second and fourth columns of Table 1.

From the results it is evident that the T-pattern-based approaches perform better than compression-based approaches. It transpires that Magnusson’s original scheme produces too many (spurious) T-patterns making high-confidence prediction impossible. This is most apparent in the 2-person scenario where the intermingling of 1-person patterns generates a large number of new combinations, a fair bit of which are erroneously identified as T-patterns. The GMM approach fares much better, even in the more difficult 2-person scenario.

### 5.2. The MERL Motion Detector Dataset

We have used the MERL motion detector dataset for a larger scale experiments [3]. The T-pattern approach is not applicable to this dataset in its original form, because the number of unique events (*i.e.*, number of sensors) and the sequence length are both much longer than it was for the original scenarios considered by Magnusson.

The MERL dataset consists of activations recorded from more than 150 passive infrared (PIR) motion detectors placed around the MERL research facility over a large period of time. The PIR sensors fire when someone (or something) passes near the sensor. Via simple binary activations of these sensors, this dataset expresses the residual trace of the activity of all people working in the two-floor facility. It has been previously used in the IEEE Information Visualization Challenge, and presents a significant challenge for behavior analysis, search, manipulation and visualization. The accompanying ground truth contains partial tracks and behavior detections, as well as map data and anonymous calendar data. We have two separate experimental setups on this dataset.

Our first experiment considers 15 sensors and contrasts GMMTPat with the two TPattern variants we introduced before (TTPat and SITPat). We use a small portion of the MERL data for this purpose, as the temporal requirement for the TPattern variants are prohibitive. 5-fold validation is used to report the results in this section, with non-overlapping folds. The 15 sensors are selected as five clusters of sensor triplets, where each triplet is in close proximity and highly correlated, but the clusters are remotely located in the building, thus uncorrelated in principle. Any correctly sequenced within-cluster patterns are *correct*, and any cross-cluster patterns are *spurious*. Out-of-sequence patterns within clusters can also be detected. If a corridor has sensors A, B and C in a sequence, the pattern A-C-B is an example to these. This type of patterns are in a gray area; if they are not found, they won’t be missed, and if they are found, they won’t hurt. We labeled them as *gray*. We report the number of correctly found, missed, spurious and gray area patterns separately for each method.

We also consider Bonferroni correction in this section. The number of tests needs to be estimated for Bonferroni correction. The number of tests per event pair was elaborated before, we now complement this with the estimation of the number of event pairs. If there are *n* sensors, the number of elementary patterns we need to consider is *n*^2^. Assume *m* of these are accepted as patterns. The number of tertiary patterns to be examined will be *nm*. In this section we will not go beyond tertiary patterns; they are sufficient for comparison purposes. Assume a topologically uniform placement of sensors, in a simple mesh. Each sensor will have four neighbours, with which it would form elementary patterns, creating 2*n* patterns in the process (ignoring boundary conditions). Adding the self-loop, which is often encountered, we estimate *m* = 3*n*. Thus, the estimated number of independence tests are *N_SIT Pat_* = 8 log_2_(*h*)*n*^2^, *N_TT Pat_* = 6*hn*^2^, and *N_GMMT Pat_* = 4*n*^2^. The independence testing for GMMTPat further reduces this number, as we no longer test all pairs of events for the existence of T-patterns. For the original T-pattern algorithm, on the other hand, this number would be *N*_*Tpat*_ = 2*h*(*h* + 1)*n*^2^. For an event horizon of 300 steps and 150 sensors, this means four billion tests.

Table 2 shows the average pattern counts and numbers of tests obtained with all three methods, as well as their Bonferroni-adjusted variants. The last row displays the expected number of tests, computed under the mentioned assumptions. The Bonferroni adjustment is largely robust to changes in this value, as long as the fluctuation is under an order of magnitude. It is clear that while Bonferroni adjustment eliminates many spurious patterns, it does not effect the time complexity much. There is of course a gain due to eliminated patterns; less patterns are tested in the end. But GMMTPat is much more efficient than SITPat and TTPat, which in turn are much more efficient than the original T-Pattern scheme.

In the final experiment, we prediction and Voronoi graph construction with GMMTPat on a large portion of the MERL set. We have used the recorded sensor events between 21 March 2006 and 11 June 2006 for training, and there are four and a half million events in this subset, generated by 154 sensors. As the test set, we use a different set of recordings, collected a year later (May 24, 2007–July 2, 2007), comprising about two million events. Due to the large number of available instances, cross-validation was not used in this study.

The complete motion ground truth for people using the environment is not available, as the sensor outputs are sometimes ambiguous. Furthermore, it is not possible to have rapid activations from a single sensor in succession, and some activity is lost. Finally, the network transmission of the events from sensors to the central recording server is reported to cause minor data loss from time to time. Along with sensor activations, some information about movements called *tracklets* are provided. Each tracklet is a directed graph of sensor activations, which possibly belongs to a single person. The MERL dataset was investigated in [37], who proposed a number of interaction features for sampling-based analysis of massive datasets. Entropy is proposed as a robust measure (suitable for cases where data are abundant) to assess relative organization of event distributions.

Since the amount of data is massive, we do not construct the whole cascade of T-patterns, but look at the elementary patterns, each composed of two basic sensor events spaced at most five minutes apart. For each such pattern, the potential critical interval is found by fitting a two-component Gaussian with the EM algorithm to the pooled interval times between the sensor firings, as described. Since the data are 1-dimensional, the convergence is fast (less than 10 iterations) and robust. Our experiments show that using more than 5,000 events for a single candidate pattern is not beneficial, as the distribution is very well approximated with 5,000 events. For real patterns, the first Gaussian has a very narrow shape that is characterized by a small standard deviation in comparison to the second Gaussian.

**Figure 4.**
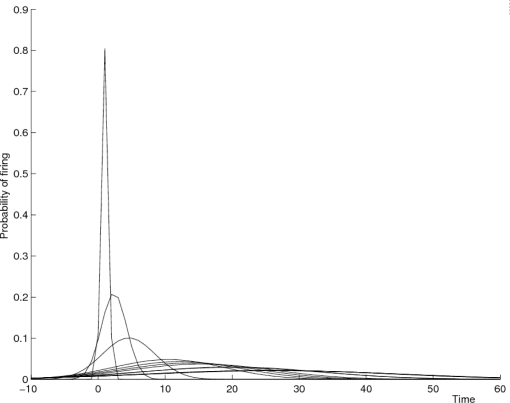
The Gaussian peaks for successive sensor firings after a given sensor event on a corridor.

Figure 4 shows the first Gaussian peak for a number of subsequent sensors along a corridor coupled with the first sensor in the corridor. The origin denotes the firings of the first sensor. The second sensor activation is very salient, producing a clear peak near zero. The third sensor (again coupled with the first sensor in the corridor) produces a smaller peak, and moves away from the first event in time. As we move on to sensors along the corridor, the peaks get flatter. The data range is 300 seconds, and the second peak for each distribution is usually between 120 and 180 seconds, *i.e.*, out of the figure.

We use the elementary patterns detected by the algorithm to construct a Voronoi graph, which reflects the topology of the environment. Technically, the Voronoi graph or the Voronoi diagram of an environment is made up of points equidistant to existing obstacles, and thus serves as a roadmap [38,39]. This is a useful representation if the exact positions of the wireless sensors and the map of the environment are missing, typically in scenarios where the deployment is fast and requires minimum manual intervention.

**Figure 5.**
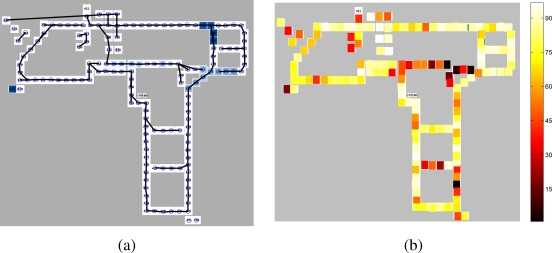
**(a)** Automatically generated Voronoi graph of the MERL lab, superposed on the layout. The circles indicate approximate sensor locations, and the connection links indicate the elementary patterns. **(b)** Predictability of the next sensor event, given the activation of a sensor. Activation of sensors shown with light colours is a good predictor of the next related event.

In our implementation, every sensor is shown as a node in this graph, and once the elementary T-patterns [*i*, *j*] are found, they are simply joined by an edge. For a better visualization, we used the following pruning process. Assume node *i* and node *j* are connected with a T-pattern. The edge that represents this pattern is pruned, if there exists a node *k* that has stronger T-patterns to both node *i* and node *j*. The strength of the pattern is reflected by the likelihood ratio of the means of the two Gaussians that model the inter-event times. A higher ratio is indicative of a stronger peak, and consequently, a stronger relation. The pruning process considers patterns sequentially, sorted by ascending pattern strength. Figure 5(a) shows a map of the environment and the superposed Voronoi graph.

By using the T-patterns, we can try to predict events based on the activation of a given sensor. This is actually more powerful than predicting the next event in the system, as we can give a temporal window (*i.e.*, the critical interval) to indicate when an expected event shall occur. From the learned set of patterns, we select the two strongest T-patterns for each sensor *i*.

For each sensor activation of the test set, we looked at the two best T-patterns, and checked the corresponding critical intervals (given by two standard deviations) for the expected events. If at least one event was detected, the prediction was counted as a success. As the number of sensors increased in time, we did not take into account activations from sensors that were missing in the training data. The prediction accuracy under this protocol was 75%.

It is also possible to analyse the prediction success sensor by sensor. Figure 5 (b) links prediction success to the sensor locations. For some locations (e.g., long corridors) the prediction accuracy is very high (up to 94 per cent), whereas predictability drops near junctions.

## 6. Conclusions

Recent progress in sensor technology makes it necessary to create algorithms that are capable of discovering structure in large-scale and possibly heterogeneous sensor systems. In this paper we have reviewed existing methodologies for the discovery of temporal patterns in sensor data. We have explicitly contrasted compression-based methods, which collapse the sequence into a string and then extract repetitive “words”, with the T-pattern approach, which takes advantage of the time dimension to find the typical delay between related events. We have proposed two improvements to the basic T-pattern methodology (referred to in this text as GMM T-patterns) that significantly improve the performance. Experiments show that T-patterns outperform the compression-based techniques and the proposed improvements (independence testing and GMM-modelling of correlation times) yield more reliable results.

We have applied the modified T-pattern algorithm on a recently published challenging dataset, consisting of binary motion sensor activations. We have shown that the proposed GMMTPat method significantly reduces the temporal complexity, even when contrasted to variants of T-pattern approach that are several orders of magnitude faster than the original. We have shown the effect of Bonferroni adjustment in eliminating spurious patterns. We have also assessed the prediction accuracy, in which the detected patterns are used to predict the firing of the next sensor in the pattern, and automatic construction of the Voronoi graph, which is a proximity-based physical map of the environment. We have validated the latter visually, by superposing it on the map of the environment that shows the true locations of the sensors. As a result, we have shown that the proposed method can be used for predicting events, or discovering the layout from the simple sensor activation patterns. The proposed method is not particular to motion sensors, and can be extended to any sensor activity where discrete events can be identified.

The application of data-mining methods to this problem seems very promising, and is conceived as a future work. In particular, the WINEPI algorithm [27] and its extensions have a similar formulation with the T-pattern algorithm, and the two approaches can be contrasted for their merits and drawbacks in real and simulated data.

## Figures and Tables

**Table 1.** Percentage correct predictions at the 20% confidence level. Due to inherent randomness, the prediction upper-bound is 70%.

	Layout 1		Layout 2	

	1 person	2 persons	1 person	2 persons
LZ	29.8	17.7	56.5	13.2
ALZ	21.1	18.8	66.4	19.6
LZW	28.9	22.0	60.5	15.1
T-patterns	28.8	17.1	61.5	24.2
GMM T-patterns	34.8	29.3	61.9	48.3

**Table 2.** Comparative evaluation of GMMTPat method.

	Without Bonferroni			With Bonferroni		

	SITPat	TTPat	GMMTPat	SITPat	TTPat	GMMTPat
Spurious	111.6	86.6	1.4	0.0	2.0	0.0
Correct	85.8	94.2	47.6	61.2	74.8	37.6
Missed	29.2	20.8	67.4	53.8	40.2	77.4
Gray	30.2	31.8	6.6	17.2	20.4	3.4

*α*	462.910	17.180	690	363.740	12.790	635
*E*[*α*]	405.000	14.812	900	405.000	14.812	900
